# What is the “weight” of body mass index on sexual functioning in women? A mediation model

**DOI:** 10.1007/s40519-020-00995-4

**Published:** 2020-09-12

**Authors:** Maria Di Nardo, Chiara Conti, Giulia Di Francesco, Giulia Nicolardi, Maria Teresa Guagnano, Piero Porcelli

**Affiliations:** 1 Department of Psychological, Health and Territorial Sciences, University “G. d’Annunzio” of Chieti‐Pescara, Via dei Vestini 31, 66100 Chieti, Italy; 2 Department of Medicine and Aging, University “G. d’Annunzio” Chieti-Pescara, Chieti, Italy

**Keywords:** BMI, Body Image, Self-esteem, Female sexual functioning, Obesity

## Abstract

**Purpose:**

It is well known that body mass index (BMI) affects how individuals perceive their well-being and that obese individuals tend to report poorer levels of subjective health status. The aim of this study was to compare subjects with and without FSD and to examine the direct and indirect impact of BMI on female sexual dysfunction (FSD) in overweight/obese and normal-weight women.

**Methods:**

A cross-sectional study was conducted on 186 overweight/obese and 233 normal-weight women. FSD assessed with the Female Sexual Function Index (FSFI) was investigated in relation to body satisfaction assessed with the Body Uneasiness Test (BUT-A) and self-esteem assessed with the Rosenberg Self-Esteem scale (RSE).

**Results:**

No difference in the prevalence of FSD was found between overweight/obese (44.4%) and normal-weight women (55.6%), even though significant between-group differences in body image were found. Structural Equation Modelling (SEM) showed that BMI contribute to FSD only through the mediating role of body dissatisfaction and self-esteem.

**Conclusions:**

The present results support the notion that sexual functioning is not related directly to BMI in women but to a more complex interactions of body weight, satisfaction with one’s own body image, and levels of self-esteem. Clinicians should take into account that for women having a good sexual life seems not related to body weight but to the way their body weight is perceived within the context of self-image.

**Level of evidence:**

Level III, case–control analytic study.

## Introduction

Female sexual dysfunction (FSD) is a multifactorial sexual health problem that affects a substantial number of women worldwide [1] with a prevalence between 25 and 63% [2]. FSD is characterized by disturbances in the psychophysiological changes associated with the sexual response cycle [3].

Personality dimensions as neuroticism, introversion, and low positive affectivity could play a role in predisposing the development of sexual dysfunction [4]. Neuroticism has been related to global sexual functioning [5], sexual arousal [6], and orgasmic difficulties [6, 7] in women. Depressive symptoms are associated with impairments in sexual function and sexual dissatisfaction [8]. Women with depressive symptoms are significantly more likely to report problems in sexual arousal, physical pleasure and emotional satisfaction [9]. In the most recently released edition of the Diagnostic and Statistical Manual of Mental Disorders, 5th Edition (DSM-5) [10], FSD entails the following disorders: sexual interest/arousal disorder, female orgasmic disorder, and genito-pelvic pain/penetration disorder. The new DSM-5 deeply revised DSM-IV-TR [11] criteria that classified FSD on a linear model of the human sexual response cycle consisting of successive stages of desire, arousal, and orgasm [12–14] whereas there are consolidated data today to claim that the interplay between motivation, arousal, and pleasure is more complex than previously thought [15].

Sexual difficulties in women are greatly influenced by both health related and psychosocial factors, and are associated with low quality of life and impaired interpersonal relationships [16]. Given the close psychological and somatic relationships embedded in the expression of human sexuality, it is not surprising that both psychological and medical conditions may manifest as sexual impairment [17]. For example, although breast cancer itself is not associated with sexual dysfunction, surgical, radiological, and hormonal treatments of breast cancer are associated with them. Women who underwent radical mastectomy had greater sexual dysfunction than those who received conservative breast therapy [18]. Sexual functioning is affected in patients with diabetes because of reduced energy from suboptimal glycemic control, alteration of self-image from obesity related to insulin resistance, and interpersonal difficulties deriving from the management of the illness (e.g., dietary control and monitoring glucose) [19, 20]. In addition, body dissatisfaction that can lead to choose the kind of treatment for obesity, may affect sexual functioning. For example, obese adults seeking bariatric surgery had worse quality of sexual life than those not seeking surgical treatment, even after controlling for body mass index (BMI) [21–24] as well as lower self-esteem and quality of life and higher depression, anxiety, and stress [25].

Psychiatric disease is among the most important risk factors for women’s sexual dysfunction [26–29]. Women with schizophrenia and schizophrenia spectrum disorders reported a very high burden of sexual dysfunction, with 60–80% of women being affected [30–35]. In addition, antipsychotic medications, symptoms of psychosis, institutionalization and societal stigma are all likely contributory factors [36].

Sexual dysfunction may also be related to obesity, even though the relationship between body weight and sexual functioning is poorly studied [37, 38] and, therefore, not fully understood. For example, it is not clear whether obesity increases directly the risk of sexual problems or whether its effects on sexual function are mediated through health related and psychosocial factors [39]. In investigating the link between body weight and sexual dysfunctions, gender also has an impact. In men, the link between obesity and erectile dysfunction is widely investigated and largely supported by cross-sectional and prospective studies [37, 40, 41]. Conversely, in women the relationship between sexual function and overweight/obesity is still largely unclear [39, 42–45]. Some studies showed that female obesity is associated with specific sexual dysfunctions such as loss of desire, poor sexual function, lack of interest in a sexual relationship, and higher sexual dissatisfaction [46–48]. Obese women frequently experience low self-esteem, poor self-acceptance of body image, and difficulty in interpersonal relationships, all of which likely interfere with the quality of sexual life [49]. Further evidence demonstrates the link between obesity and low self-esteem, in certain female population cohorts [50, 51]. Unfortunately, to the best of our knowledge, no research has investigated the relationship between self-esteem, body satisfaction and female sexual functioning in more depth. Some investigations pointed out that the higher the individual’s body satisfaction, the higher the perceived quality of sexual life. For example, Asfhari et al. demonstrated that women with a positive body image had higher sexual function perception compared to women with a negative body image. In other words, sexual desire, arousal, and orgasm are suggested to improve with increased body image perception [52]. In contrast, however, other studies have suggested that female obesity does not seem to be a major contributor to sexual dysfunction [53, 54]. For example, Kadioglu et al. found no association between impaired sexual functioning, obesity, and metabolic syndrome when compared with a control group, even though vaginal lubrication was affected by metabolic syndrome [53]. In a study that was aimed to identify the frequency of FSD among obese and overweight women, FSFI scores were not correlated with any of the anthropometric measurements (body mass index (BMI), waist-to-hip ratio (WHR) and fat percent) [54]. In sum, research data suggest that the relationship between sexual functioning and obesity in women is highly complex and influenced by multiple factors, including psychological distress [55, 56], low self-esteem [57, 58], and body image dissatisfaction [59].

In this study, we aimed to investigate to which extent self-image, conceived as the composite of body image and self-esteem, is related to BMI and sexual functioning in a sample of normal-weight and obese/overweight women. In particular, based on previous literature, we expected that (a) more FSD would be exhibited by overweight/obese women with higher body dissatisfaction and lower self-esteem than normal-weight women, and (b) BMI would affect FSD through the mediating role of body image and self-esteem rather than directly.

## Materials and methods

### Participants

The clinical sample was constituted by consecutive female adult outpatients meeting World Health Organization (WHO) criteria for overweight and obese (see below) and referred to the Obesity Outpatients Centre at the University Clinical Hospital of Chieti, Italy, between 2017 and 2018. Of the 223 recruited women, 186 (82%) accepted to be enrolled. Main reason for not participating was lack of time. The control subjects included 233 adult women who were recruited among female outpatients referred to general practitioners in the same geographic catchment area. Control subjects were screened out for exclusion criteria (see below) and screened in if their body mass index (BMI) was within the normal range between 18 and 25 kg/m^2^. For inclusion, all participants were required to self-report as-usual sexual activities during the last 4 weeks and to complete a questionnaire on age, education, body weight, height, marital status, presence of a stable romantic relationship, diet, pharmacotherapy, age of menarche, use of hormonal contraception, and menopausal status. To optimize ecological validity, all subjects aged from 20 to 48 years were included. Exclusion criteria were current breast-feeding, pregnancy, post-partum period, non-Italian speaking, severe medical comorbidity, mental retardation, current or past diagnosis of psychotic disorders, eating disorders and substance or alcohol abuse.

All subjects provided written informed consent to take part in the study. The study was reviewed and approved by the Ethics Committee of University G. d’Annunzio - Chieti-Pescara and carried out in accordance with the World Medical Association Declaration of Helsinki and its subsequent revisions.

### Measures

#### Body mass index

BMI was calculated as weight in kilograms divided by the square of height in meters (kg/m^2^), and indicating nutritional status in adults. The BMI limits are age- and gender-independent and WHO has classified BMI-related groups in adults: BMI ≤ 18.5 kg/m^2^, underweight; 18.5–24.9 kg/m^2^, normal weight; 25–29.9 kg/m^2^, overweight; 30–34.9 kg/m^2^, class 1 obesity; 35–39.9 kg/m^2^, class 2 obesity; and ≥ 40 kg/m^2^, class 3 obesity.

#### Sexual functioning

The Italian version of the 19-item Female Sexual Function Index (FSFI) [60] questionnaire was employed for investigating the sexual functioning of participants during the last 4 weeks. The FSFI is a 19-item self-report instrument that was developed for evaluating key dimensions of female sexual functioning [61]. It assesses six specific domains: Sexual Desire (frequency and desire level), Arousal (frequency, level, confidence and satisfaction), Lubrication (frequency, difficulty, frequency of maintaining and difficulty in maintaining), Orgasm (frequency, difficulty and satisfaction), Satisfaction (level of closeness with a partner, sexual relationship and overall sex life), and Dyspareunia (frequency, whether during vaginal penetration and pain frequency following vaginal penetration). Each domain is scored on a 7-point Likert scale from 0 to 6, with a higher score indicating better function. A score is calculated for each of the six domains, and the total score is obtained by summing of the six subscale scores. A score of 26.55 or lower indicates the presence of sexual dysfunction. For this sample, Cronbach’s *α* was 0.99 for the total scale, 0.86 for the sexual desire, 0.97 for the arousal, 0.98 for the lubrication, 0.96 for the orgasm, and 0.97 for the pain, and 0.98 for the satisfaction dimensions.

#### Body image

The Body Uneasiness Test (BUT) is a self-report questionnaire specifically designed to explore several areas of body image in clinical and non-clinical populations as body shape and/or weight dissatisfaction, avoidance, compulsive control behaviors, detachment and estrangement feelings towards one’s own body, and specific worries about particular body parts, shapes, or functions [59].

The term “body uneasiness” is used to describe not only body dissatisfaction but also associated emotions, such as anxiety, alarm, trepidation, worry, mistrust, misgiving, doubt, suspicion, and embarrassment. The BUT consists of two parts: (1) BUT-A consists of 34 items with a score ranging from 0 (*never*) to 5 (*always*). The scores are combined in a Global Severity Index (GSI) and in 5 subscales resulting from factorial analyses: Weight Phobia (WP), Body Image Concerns (BIC), Avoidance (A), Compulsive Self–Monitoring (CSM), and Depersonalization (D); 2) BUT-B has 37 items a ranged in 8 factors evaluating specific worries about body parts or functions, item examples are “Of my body, in particular, I hate”. The responses are scored on a 6-point Likert scale, ranging from 0 (*never*) to 5 (*always*). For the aim of the present study, we used only part A. Higher score indicates higher dissatisfaction with one’s own body image. For this sample, Cronbach’s *α* was 0.96 for the total scale GSI, 0.89 for the WP, 0.92 for the BIC, 0.87 for the A, 0.74 for the CSM, and 0.85 for the D dimensions.

#### Self-Esteem

The Rosenberg’s Self-Esteem Scale includes 10 items asking participants to rate their feelings about themselves [62]. Five items are positively worded and five negatively worded. Item examples are “I feel that I have a number of good qualities”, and “At times I feel that I’m no good at all”. The responses are scored on a 4-point Likert scale, ranging from 0 (*strongly disagree*) to 3 (*strongly agree*). Self-esteem scores are calculated as sum scores, and the possible range of scores is 0–30. A higher score indicated a stronger sense of self-esteem. For this sample, Cronbach’s *α* was 0.84.

### Statistical analyses

Statistical analysis was carried out using IBM SPSS 23.0 for Windows (IBM Corp, Armonk, NY, USA, 2015) and STATA 13.

A two-step strategy was used for data analysis.

First, socio-demographic and clinical variables between overweight and obese women and control group were compared using Student’s *t* test. The standardized mean difference was used as a measure of effect size. A standardized effect size (Cohen’s *d*) of 0.20–0.50 is considered small, 0.50–0.80 moderate, and > 0.80 large [63].

Second, Structural Equation Modelling (SEM) was used to assess the direct and indirect effects of BMI on FSD through the mediating role of body image and self-esteem. SEM is a set of statistical techniques used to measure and analyse the relationships of observed and latent variables. It examines linear causal relationships among variables, while simultaneously accounting for measurement error. SEM can be viewed as a combination of confirmatory factor analysis and regression or path analysis. Latent variables or factors are considered to represent theoretical constructs that can be interpreted as latent traits underlying the measured items and inducing dependence among them. Observable variables or factors are variables that can be observed and directly measured [64–66].

The analysis of the hypothesized mediation model was based on the two-step procedure: in the first step, confirmatory factor analysis was used to construct a measurement model. In the second step, the established structural model was verified. The evaluation of model fit was based on Chi squared plus recommended criteria for a set of fit indices. Comparative Fit Index (CFI) and Tucker Lewis Index (TLI) = 0.95, which indicate a reasonable fit of the model [64, 67–69]. Root Mean Square Error of Approximation (RMSEA) of 0.05 can be considered as a good fit; values between 0.05 and 0.08 indicate acceptable fit [67, 70, 71].

Missing data were replaced by way of multiple imputation algorithms. SEM, with a maximum likelihood estimation method, was used to evaluate the fit of the hypothesized model based on the following multiple criteria: Chi squared (*χ*^2^) (*p* value > 0.05), RMSEA close to 0.06 or less for a well-fitted model, CFI near 0.90 or greater and TLI near 0.90 or greater [69].

The structural components of the model included one exogenous observed variable for the BMI, one endogenous latent factor for the FSFI, and two continuous mediator variables (both endogenous and exogenous). Hypotheses regarding the structural relationships among the constructs in the final model were evaluated using the magnitude of path coefficients (standardized coefficient) and their significance [64].

## Results

### Characteristics of the participants

Table 1 reports the socio‐demographic and clinical characteristics of the total sample.

**Table 1:** Comparisons of socio-demographic and clinical characteristics between overweight/obese women and normal weight women

Variables	Total sample (*N* = 419)	Overweight and obese (*N* = 186) (44.4%)	Normal weight (*N* = 233) (55.6%)	*t*	*p*	*d*
Age (M ± SD)	27.01 ± 7.45	28.43 ± 8.77	25.88 ± 6.03	3	< 0.001	0.35
Education						
Elementary School	4 (1.1)	4 (1.1)				
Secondary School	28 (6.7)	20 (10.7)	9 (4.0)			
High School	212 (50.6)	89 (48)	123 (57)			
Bachelor’s degree	158 (37.7)	75 (40.1)	91 (39.0)			
Marital status						
Unmarried	315 (81.2)	118 (92)	194 (68)			
Married	57 (14.7)	41 (24.0)	15 (7.0)			
Separated/divorced	10 (2.6)	8 (5.1)	1 (0.5)			
Cohabitant	6 (1.4)	5 (2.9)	1 (0.5)			
BMI (M ± SD)	25.59 ± 5.92	30.94 ± 4.84	21.33 ± 1.33	3	< 0.001	0.29
BUT GSI (M ± SD)	1.22 ± .93	1.46 ± 1.00	1.03 ± .83	4.86	< 0.001	0.47
WP (M ± SD)	1.75 ± 1.20	1.97 ± 1.27	1.57 ± 1.11	3.45	< 0.001	0.33
BIC (M ± SD)	1.53 ± 1.19	1.93 ± 1.31	1.21 ± .98	6.37	< 0.001	0.63
A (M ± SD)	0.60 ± 0.88	0.83 ± 0.99	0.41 ± 0.73	4.96	< 0.001	0.49
CSM (M ± SD)	1.11 ± .91	1.23 ± 1.30	1.07 ± 0.92	1.45	< 0.001	0.14
D (M ± SD)	0.74 ± 0.92	0.92 ± 1.02	0.60 ± 0.80	3.55	< 0.001	0.35
FSFI (M ± SD)	27.55 ± 5.55	27.30 ± 5.60	27.74 ± 5.52	0.81	0.56	0.07
Desire (M ± SD)	4.03 ± 1.06	4.04 ± 1.12	4.01 ± 1.02	0.32	0.14	0.28
Arousal (M ± SD)	4.43 ± 1.16	4.35 ± 1.20	4.49 ± 1.13	0.12	0.51	0.12
Lubrication (M ± SD)	4.81 ± 1.21	4.82 ± 1.17	4.81 ± 1.25	0.07	0.43	0.08
Orgasm (M ± SD)	4.47 ± 1.33	4.42 ± 1.33	4.50 ± 1.34	0.71	0.58	0.60
Satisfaction (M ± SD)	4.86 ± 1.30	4.79 ± 1.25	4.93 ± 1.34	0.15	10.08	0.10
Pain (M ± SD)	4.92 ± 1.12	4.88 ± 1.21	4.98 ± 1.05	0.05	0.53	0.08
RSE (M ± SD)	29.14 ± 5.07	29.03 ± 5.07	29.23 ± 5.09	0.03	0.41	0.03

*BMI* body mass index, *BUT GSI* Body Uneasiness Test Global Severity Index, *WP* weight phobia, *BIC* body image concerns, *A* avoidance, *CSM* control self-monitoring, *D* depersonalization, *FSFI* Female Sexual Function Index, *RSE* Rosenberg’s Self-Esteem Scale

The overall 419 participants (*n  *= 186, 44.4%, in the obese/overweight sample and *n * = 233, 55.6%, in the normal-weight sample) reported a mean age of 27.04 ± 7.70. Most of the participants had graduated from high school and bachelor’s degree (*n  *= 215, 50.6%, and *n * = 158, 37.7%, respectively), and were unmarried (*n  *= 315, 81.2%). No between-group difference was found for any socio-demographic variable.

### Differences between groups

As shown in Table 1, the two groups of subjects were remarkably similar when they were evaluated for sexual functioning and self-esteem. No statistical differences were observed between the two groups for FSFI total score (*d * = 0.07) and each domain score such as desire (*d * = 0.28), arousal (*d * = 0.12), lubrication (*d * = 0.08), orgasm (*d * = 0.60), satisfaction (*d * = 0.10), and pain (*d * = 0.08), respectively.

According to the total FSFI score, FSD was found in 81 (43.5%) overweight/obese subjects and in 77 (33%) control subjects with no between-group difference (*t  *= 2.18, *p = *0.02) and in 72 (40.2%) subjects with a stable relationship and in 107 (59.7%) subjects without a stable relationship. No differences were also found for self-esteem (RSE score, *p = *0.41). A small difference was found in overweight/obese women than control group about age (*d* = 0.35). In addition, overweight/obese women showed greater body dissatisfaction than the control group in several domains of BUT such as total score (BUT-GSI, *d * = 0.47) and body image concerns (BIC, *d  *= 0.63) in the moderate effect-size range, weight phobia (WP, *d * = 0.33), avoidance (A, *d* = 0.49), depersonalization (D, *d* = 0.35) in the small range of effect size, and, at a lesser extent, control self-monitoring (CSM, *d  *= 0.14) scores.

Table 2 reports the comparisons of age, BMI and psychological variables between subjects with (*n * = 158, 37.4%) and without FSD (*n * = 261, 62.6%).

**Table 2:** Comparisons between subjects with and without female sexual dysfunction

Variables	Total sample (*N* = 419)	FSD (*N* = 158) (37.4%)	No FSD (*N* = 261) (62.6%)	*t*	*p*	*d*
Age (M ± SD)	27.08 ± 7.83	27.77 ± 8.68	26.53 ± 7.04	1.57	0.063	0.16
BMI (M ± SD)	25.59 ± 5.92	26.22 ± 5.45	25.11 ± 6.01	1.52	0.21	0.19
BUT GSI (M ± SD)	1.22 ± 0.93	1.38 ± 0.97	1.11 ± .89	2.89	0.004	0.29
WP (M ± SD)	1.75 ± 1.20	1.89 ± 1.21	1.65 ± 1.18	1.97	0.049	0.20
BIC (M ± SD)	1.53 ± 1.19	1.72 ± 1.17	1.40 ± 1.17	2.67	0.008	0.27
A (M ± SD)	0.60 ± 0.88	0.82 ± 0.96	0.45 ± 0.78	4.15	< 0.001	0.43
CSM (M ± SD)	1.11 ± 0.91	1.21 ± 1.01	1.04 ± .84	1.83	0.067	0.18
D (M ± SD)	0.74 ± 0.92	0.87 ± 0.98	0.65 ± 0.86	2.30	0.022	0.24
RSE (M ± SD)	29.14 ± 5.07	27.69 ± 5.23	30.00 ± 4.78	4.56	< 0.001	0.46

*FSD* Female Sexual Dysfunction, *BMI* body mass index, *BUT GSI* Body Uneasiness Test Global Severity Index, *WP* weight phobia, *BIC* body image concerns, *A* avoidance, *CSM* control self-monitoring, *D* depersonalization, *RSE* Rosenberg’s Self-Esteem Scale

The two groups of women were remarkably similar when they were evaluated for age (*d *= 0.16) and BMI (*d *= 0.19). In contrast, women with FSD showed greater body dissatisfaction than those without FSD in several domains of BUT, particularly higher Avoidance (A, *d *= 0.43) in the moderate range of effect size and higher weight phobia (WP, *d *= 0.20), body image concerns (BIC, *d *= 0.27), and depersonalization (D, *d *= 0.24) in the small effect size range. In addition, women with FSD showed lower self-esteem than the controls (*d *= 0.43) with effect size in the moderate range.

### Structural equation model

**Fig. 1:**
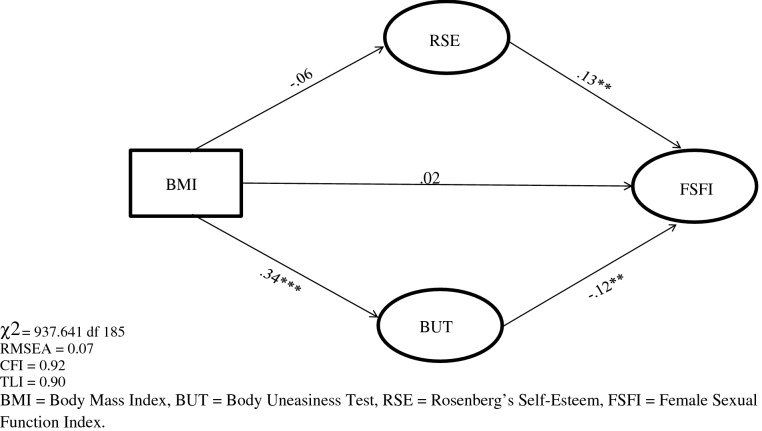
Structural equation modelling among BMI, RSE, BUT and FSFI

SEM was used to evaluate the direct and indirect effects of BMI on female sexual dysfunction through the mediating role of body image and self-esteem. The structural components of the model included one exogenous observed variable for the BMI, one endogenous latent factor for the FSFI, and two continuous mediator variables (both endogenous and exogenous). Table 3 and Fig. 1 show the direct and indirect effects of the model and the path analysis of the parameter estimates respectively. All the observed variables were loaded on their corresponding latent constructs, supporting the validity of the construct of each latent construct, and standardized residuals were normally distributed. The parameter model estimates indicated that there were no significant direct effects of BMI on FSFI, whereas there was a significant, direct, and positive effect of RSES on FSFI (*β  *= 0.13) and a significant, direct, and negative effect of BUT on FSFI (*β  *= − 0.12). In addition, a significant direct effect of BMI was found on BUT (*β  *= 0.34), whereas there was no significant direct effect of BMI on RSES. Put in more clinically-bound words, results of SEM indicate that, (a) the higher self-esteem level, the greater the increase of sexual functioning; (b) the higher the increase of body image dissatisfaction, the lower the level of sexual functioning. Finally, a negative and indirect effect of BMI on FSFI through the mediation of RSES and BUT was reported (*β*^indirect^= − 0.05, *p* < 0.01).

**Table 3:** Effects of exogenous constructs in model

Exogenous variables	Endogenous variables	*β*	*z*	*R*^2^	Direct effects	Indirect effects
BMI	RSE	− 0.06	1.20	0.01	− 0.06	
BMI	BUT	0.34	3.94	0.20	0.34***	
BMI	FSFI	0.02	2.50	0.18	0.02	− 0.05**
RSE		0.13	2.38	0.13**		
BUT		− 0.12	2.24	− 0.12**		

** p < .01, *** p < .001

*BMI* body mass index, *RSE* Rosenberg’s Self-Esteem, *BUT* Body Uneasiness Test, *FSFI* Female Sexual Function Index

The values of multiple fit indices indicated that the proposed model provided acceptable fit data, *χ*^2^ = 937.641, *df *= 185, *p* < 0.001, TLI = 0.90, CFI = 0.92, and RMSEA = 0.07.

In sum, the mediation model analysis showed that BMI affected sexual functioning not directly but only through the mediational role of variables related to body image and body concerns as self-esteem and body uneasiness.

## Discussion

The present study aimed at investigating the relationship between female sexual functioning and BMI. Few studies are available to date and their results are controversial. The association between obesity and sexual dysfunction in women has been supported by some findings [2, 47, 72] but not [53, 54] or only partially [46] in others. In our study, we added to the state of the art by investigating the extent to which the wider concept of self-image, conceived as the composite of body image and self-esteem, may mediate the relationship between BMI and female sexual functioning in overweight and obesity conditions compared with normal-weight women.

In our first hypothesis, we expected that overweight and obese women would exhibit lower levels of self-esteem and higher levels of FSD and body dissatisfaction compared to controls. This hypothesis was partially confirmed. Through the BUT scales, overweight and obese women reported higher levels of fear of being or becoming fat (WP), over-concern with physical appearance (BIC), compulsive checking of physical appearance (CSM), and overall severity of body image impairment compared with normal-weight women. This finding is consistent with literature showing that body dissatisfaction is greater in obese subjects than normal-weight subjects [73], that high degree of adiposity increases the disturbances of the body image, and that the body mass correlates positively with non-acceptance [74]. However, our findings could not confirm the expectation that sexual functioning is affected by overweight/obese women. Data on the association between body weight and sexual functioning have not received full confirmation from literature. Although some previous investigations reported such association [e.g., 21, 46, 48, 75], other studies showed no difference in good sexual functioning between obese and normal-weight women [76–78]. Some hypothesis can be made to explain the controversial results. For example, cultural differences and social desirability might influence individual attitudes to live and communicate details of intimate sexual life. In addition, it is possible that a sort of “threshold effect” could bias the different findings among studies that distinguish or collapse obese and overweight subjects. In fact, once BMI is taken into consideration, also the differences in sexual functioning between obese and normal-weight women tend to disappear [46, 48, 75]. Finally, in line with our results, several investigations pointed out to the necessity of considering psychological mediators when the relationship between BMI and female sexual functioning is evaluated [12, 46, 53, 79].

In our second hypothesis, BMI was expected to affect FSD through the mediating role of body image and self-esteem rather than directly. As expected, SEM analysis revealed no direct effect of BMI on FSD. Instead, BMI affected directly only body dissatisfaction whereas indirectly FSD through the mediating role of body dissatisfaction and self-esteem. Therefore, our results suggest that the impact of body dissatisfaction and self-esteem on female sexual functioning occurs on interactions across BMI and FSFI. This is in line with clinical experience and empirical evidence showing the importance of multiple domains of body image and self-esteem when female sexual functioning is considered, thus suggesting how feelings of self-acceptance, self-satisfaction, and positive self-evaluation are relevant in intimate relationships and sexual life of women [80]. Woertman and van den Brink showed that women with higher body satisfaction reported several positive outcomes as more frequent sexual experiences, engagement in a wider range of sexual activities, more feelings of sexual desirability, greater level of comfort with sexual activities, and fewer sexual problems than those with low body satisfaction [81]. Consistent similar association have been found in different samples of female adolescents [82], women in climacteric age [83, 84], and after bariatric surgery [44].

The results of the present study should be considered within the context of some limitations. First, the cross‐sectional study design does not allow us to determine the direction of causality between BMI, body image, self-esteem, and female sexual functioning. Low self-esteem and unsatisfaction with one’s own body image may reinforce with each other and contribute to BMI increase through the adoption of an unhealthy lifestyle, thereby leading to an overall poor psychosocial functioning, including sexual activity. A longitudinal study design could help to ascertain overtime stability of variables and to detect their causative role. Second, because the recruited sample consisted of women who declared their willingness to participate in a study about sexual functioning, the generalizability of findings may be limited. Women who volunteer to participate in sex research may be more sexually experienced, hold less traditional sexual attitudes, and report higher sexual esteem and sexual sensation seeking [85]. Third, a consecutive non‐probabilistic sample was used in this study. Future studies with probabilistic sampling procedures will be useful to investigate the involvement of BMI on FSD in overweight/obese and normal-weight women. Fourth, a number of potentially relevant factors were not controlled for, as depression and anxiety. Future investigations should take into account those factors as relevant moderating and mediating variables whose change overtime may influence the association of BMI and self-image with female sexual functioning. Finally, sexual functioning was studied with the use of self-report measures only while a multimethod procedure should be adopted by integrating self-report scale and structured interviews in future scale [60, 86–88].

In conclusion, the present study does not support the hypothesis that there is a direct association between BMI and sexual dysfunction in women. Rather, our findings showed the existence of an indirect association between BMI and the female sexual functioning through the mediating role of the self-image, composed by self-esteem and body satisfaction. Our findings offered satisfactory confirmation for the hypothesized structural model that provided an adequate fit to the data. In one word, for a woman, having a good sexual life seems not related to her body weight but to the way her body weight is perceived within the context of her self-image, that is how much she is satisfied with her body image (body satisfaction) and she appreciates herself (self-esteem). Longitudinal studies are needed to ascertain the overtime stability of these constructs and the direction of causality between factors. If confirmed, our findings may have relevant clinical implications. For example, obese/overweight women dissatisfied of their sexual activity and who attribute their poor sexual life to their BMI should be alerted that there may be personal and psychological problems underlying their poor sexual life which may be independent of body weight. In addition, programs for body weight control and the treatment of obesity should take into account that weight loss in itself might be ineffective on life satisfaction and sexual life of women. Given the relevance of sexual health on individual life, perceived poor sexual functioning may lead in turn to poor outcomes of diet and weight control intervention programs in the long run because of a vicious circle of life unsatisfaction, poor adherence to treatment, and adoption of unhealthy lifestyle behaviors [89].

***What is already known on this subject?*** It is well known that body mass index (BMI) affects how individuals perceive their well-being and that obese individuals tend to report poorer levels of subjective health status.

***What this study adds?*** Sexual functioning is not related directly to BMI in women but to a more complex interactions of body weight, satisfaction with one’s own body image, and levels of self-esteem.

## Data Availability

The datasets generated during and/or analyzed during the current study are available from the corresponding author on reasonable request.

## References

[1] Ponholzer A, Roehlich M, Racz U, Temml C, Madersbacher S (2005). Female sexual dysfunction in a healthy Austrian cohort: prevalence and risk factors. Eur Urol.

[2] Jaafarpour M, Khani A, Khajavikhan J, Suhrabi Z (2013). Female sexual dysfunction: prevalence and risk factors. J Clin Diagn Res.

[3] Basson R, Berman J, Burnett A, Derogatis L, Ferguson D, Fourcroy J, Leiblum S (2000). Report of the international consensus development conference on female sexual dysfunction: definitions and classifications. J Urol.

[4] Brotto L, Atallah S, Johnson-Agbakwu C, Rosenbaum T, Abdo C, Byers ES, Wylie K (2016). Psychological and interpersonal dimensions of sexual function and dysfunction. J Sex Med.

[5] Crisp C, Vaccaro C, Fellner A, Kleeman S, Pauls R (2015). The influence of personality and coping on female sexual function: A population survey. J Sex Med.

[6] Kennedy SH, Dickens SE, Eisfeld BS, Bagby RM (1999). Sexual dysfunction before antidepressant therapy in major depression. J Affect Disord.

[7] Harris JM, Cherkas LF, Kato BS, Heiman JR, Spector TD (2008). Normal variations in personality are associated with coital orgasmic infrequency in heterosexual women: A population-based study. J Sex Med.

[8] Baldwin DS, Manson C, Nowak M (2015). Impact of antidepressant drugs on sexual function and satisfaction. CNS Drugs.

[9] Cyranowski JM, Bromberger J, Youk A, Matthews K, Kravitz HM, Powell LH (2004). Lifetime depression history and sexual function in women at midlife. Arch Sex Behav.

[10] American Psychiatric Association (2013). Diagnostic and statistical manual of mental disorders.

[11] American Psychiatric Association (2000). Diagnostic and statistical manual of mental disorders 4th ed, Text Revised.

[12] Giles KR, McCabe MP (2009). Women’s sexual health: conceptualizing women’s sexual function: linear vs. circular models of sexual response. J Sex Med.

[13] Kaplan HS (1979). Disorders of sexual desire.

[14] Masters WH, Johnson VE, Kolodny RC (1984). Human sexuality.

[15] Clayton AH, Juarez EMV (2017). Female sexual dysfunction. *Psychiatric*. Clinics.

[16] Edwards WM, Coleman E (2004). Defining sexual health: a descriptive overview. Arch Sex Behav.

[17] Clayton A, Ramamurthy S (2008) The impact of physical illness on sexual dysfunction. In: Sexual dysfunction, olv 29, pp 70–88. Karger Publishers. 10.1159/000126625 10.1159/000126625 18391558

[18] Stead ML (2003). Sexual dysfunction after treatment for gynaecologic and breast malignancies. Curr Opin Obstet Gynecol.

[19] Bhasin S, Enzlin P, Coviello A, Basson R (2007). Sexual dysfunction in men and women with endocrine disorders. The Lancet.

[20] Rahmanian E, Salari N, Mohammadi M, Jalali R (2019). Evaluation of sexual dysfunction and female sexual dysfunction indicators in women with type 2 diabetes: a systematic review and meta-analysis. Diabetol Metab Syndr.

[21] Kolotkin RL, Binks M, Crosby RD, Ostbye T, Gress RE, Adams TD (2006). Obesity and sexual quality of life. Obesity (Silver Spring).

[22] Kolotkin RL, Crosby RD, Pendleton R, Strong M, Gress RE, Adams T (2013). Health-related quality of life in patients seeking gastric bypass surgery vs non-treatment-seeking controls. Obes Surg.

[23] Pichlerova D, Bob P, Zmolikova J, Herlesova J, Ptacek R, Laker MK, Weiss P (2019). Sexual dysfunctions in obese women before and after bariatric surgery. Med Sci Monit.

[24] Sarwer DB, Spitzer JC, Wadden TA, Rosen RC, Mitchell JE, Lancaster K, Christian NJ (2013). Sexual functioning and sex hormones in persons with extreme obesity and seeking surgical and nonsurgical weight loss. Surg Obes Relat Dis.

[25] Abilés V, Rodríguez-Ruiz S, Abilés J, Mellado C, García A, De La Cruz AP, Fernández-Santaella MC (2010). Psychological characteristics of morbidly obese candidates for bariatric surgery. Obes Surg.

[26] Avis NE, Zhao X, Johannes CB, Ory M, Brockwell S, Greendale GA (2005). Correlates of sexual function among multi-ethnic middle-aged women: results from the Study of Women’s Health Across the Nation (SWAN). Menopause.

[27] Lutfey KE, Link CL, Rosen RC, Wiegel M, McKinlay JB (2009). Prevalence and correlates of sexual activity and function in women: results from the Boston Area Community Health (BACH) Survey. Arch Sex Behav.

[28] Mitchell KR, Mercer CH, Ploubidis GB, Jones KG, Datta J, Field N, Clifton S (2013). Sexual function in Britain: findings from the third National Survey of Sexual Attitudes and Lifestyles (Natsal-3). The Lancet.

[29] Wåhlin-Jacobsen S, Kristensen E, Pedersen AT, Laessøe NC, Cohen AS, Hougaard DM, Giraldi A (2017). Androgens and psychosocial factors related to sexual dysfunctions in premenopausal women∗:∗ 2016 ISSM Female Sexual Dysfunction Prize. J Sex Med.

[30] Harley EWY, Boardman J, Craig T (2010). Sexual problems in schizophrenia: prevalence and characteristics A cross sectional survey. Soc Psychiatry Psychiatric Epidemiol.

[31] Hou CL, Zang Y, Rosen RC, Cai MY, Li Y, Jia FJ, Xiang YT (2016). Sexual dysfunction and its impact on quality of life in Chinese patients with schizophrenia treated in primary care. Compr Psychiatry.

[32] İncedere A, Küçük L (2017). Sexual life and associated factors in psychiatric patients. Sex Disabil.

[33] Kikuchi T, Iwamoto K, Sasada K, Aleksic B, Yoshida K, Ozaki N (2012). Sexual dysfunction and hyperprolactinemia in Japanese schizophrenic patients taking antipsychotics. Prog Neuropsychopharmacol Biol Psychiatry.

[34] Mahmoud SB, Zouari L, Dammak M, Thabet JB, Zouari N, Maâlej M (2013). Evaluation of sexuality in 61 subjects suffering from chronic psychosis. Sexologies.

[35] Tharoor H, Kaliappan A, Gopal S (2015). Sexual dysfunctions in schizophrenia: professionals and patients perspectives. Indian J Psychiatry.

[36] Basson R, Gilks T (2018). Women’s sexual dysfunction associated with psychiatric disorders and their treatment. Women’s Health.

[37] Esfahani SB, Pal S (2018). Obesity, mental health, and sexual dysfunction: a critical review. Health Psychol Open.

[38] World Health Organization (1997). Obesity: prevention and managing the global epidemic: report of a WHO Consultation on Obesity.

[39] Rowland DL, McNabney SM, Mann AR (2017). Sexual function, obesity, and weight loss in men and women. Sex Med Rev.

[40] Akdemir AO, Karabakan M, Aktas BK, Bozkurt A, Ozgur EG, Akdogan N, Yarıs M (2019). Visceral adiposity index is useful for evaluating obesity effect on erectile dysfunction. Andrologia.

[41] Esposito K, Giugliano D (2005). Obesity, the metabolic syndrome, and sexual dysfunction. Int J Impot Res.

[42] Brody S (2004). Slimness is associated with greater intercourse and lesser masturbation frequency. J Sex Marital Ther.

[43] Costa RM, Brody S (2016). Obesity, overweight, female sexual function, and penile-vaginal intercourse frequency. J Sex Marital Ther.

[44] Janik MR, Bielecka I, Paśnik K, Kwiatkowski A, Podgórska L (2015). Female sexual function before and after bariatric surgery: a cross-sectional study and review of literature. Obes Surg.

[45] Kirchengast S, Hartmann B, Gruber D, Huber J (1996). Decreased sexual interest and its relationship to body build in postmenopausal women. Maturitas.

[46] Esposito K, Ciotola M, Giugliano F, Bisogni C, Schisano B, Autorino R, Giugliano D (2007). Association of body weight with sexual function in women. Int J Impot Res.

[47] Mozafari M, Khajavikhan J, Jaafarpour M, Khani A, Direkvand-Moghadam A, Najafi F (2015). Association of body weight and female sexual dysfunction: a case control study. Iran Red Crescent Med J.

[48] Rabiepoor S, Khalkhali HR, Sadeghi E (2017). What kind of sexual dysfunction is most common among overweight and obese women in reproductive age?. Int J Impot Res.

[49] Kaneshiro B, Jensen JT, Carlson NE, Harvey SM, Nichols MD, Edelman AB (2008). Body mass index and sexual behavior. Obstet Gynecol.

[50] McClure AC, Tanski SE, Kingsbury J, Gerrard M, Sargent JD (2010). Characteristics associated with low self-esteem among US adolescents. Acad Pediatrics.

[51] Perrin EM, Boone-Heinonen J, Field AE, Coyne-Beasley T, Gordon-Larsen P (2010). Perception of overweight and self-esteem during adolescence. Int J Eat Disord.

[52] Afshari P, Houshyar Z, Javadifar N, Pourmotahari F, Jorfi M (2016). The relationship between body image and sexual function in middle-aged women. Electron Phys.

[53] Kadioglu P, Yetkin DO, Sanli O, Yalin AS, Onem K, Kadioglu A (2010). Obesity might not be a risk factor for female sexual dysfunction. BJU Int.

[54] Yaylali GF, Tekekoglu S, Akin F (2010). Sexual dysfunction in obese and overweight women. Int J Impot Res.

[55] Atlantis E, Baker M (2008). Obesity effects on depression: systematic review of epidemiological studies. Int J Obes (Lond).

[56] Martínez E, Gutiérrez-Bedmar M, García-Rodríguez A, Mariscal A, Muñoz-Bravo C, Navajas J (2014). Weight status and psychological distress in a Mediterranean Spanish population: a symmetric U-shaped relationship. Nutrients.

[57] Chu DT, Nguyet NTM, Nga VT, Lien NVT, Vo DD, Lien N, Van To T (2019). An -update on obesity: mental consequences and psychological interventions. Diabetes Metabo Syndr Clin Res Rev.

[58] Olchowska-Kotala A (2018). Body esteem and self-esteem in middle-aged women. J Women Aging.

[59] Cuzzolaro M, Vetrone G, Marano G, Garfinkel PE (2006). The Body Uneasiness Test (BUT): development and validation of a new body image assessment scale. Eat Weight Disord.

[60] Nappi RE, Albani F, Vaccaro P, Gardella B, Salonia A, Chiovato L, Spinillo A, Polatti F (2008). Use of the Italian translation of the Female Sexual Function Index (FSFI) in routine gynecological practice. Gynecol Endocrinol.

[61] Rosen C, Brown J, Heiman S, Leiblum C, Meston R, Shabsigh D, Ferguson R, D’Agostino R (2000). The Female Sexual Function Index (FSFI): a multidimensional self-report instrument for the assessment of female sexual function. J Sex Marital Ther.

[62] Rosenberg M (1965). Society and the adolescent self-image.

[63] Cohen J (1988). Statistical power for the behavioral sciences.

[64] Bentler PM (1990). Comparative fit indexes in structural models. Psychol Bull.

[65] Bollen KA (2002). Latent variables in psychology and the social sciences. Annu Rev Psychol.

[66] Bollen KA (1989). Structural equations with latent variables.

[67] Brown TA (2006). Confirmatory factor analysis for applied research.

[68] Kline RB (2005). Principles and practice of structural equation modeling.

[69] Schumacker RE, Lomax RG (1996). A beginner’s guide to structural equation modeling.

[70] Browne MW, Cudeck R, Bollen KA, Long JS (1993). Alternative ways of assessing model fit. Testing structural equation models.

[71] Hu L, Bentler P (1999). Cutoff criteria for fit indices in covariance structure analysis: conventional criteria versus new alternatives. Struct Equ Model.

[72] Jamali S, Zarei H, Jahromi AR (2014). The relationship between body mass index and sexual function in infertile women: a cross-sectional survey. Iran J Reprod Med.

[73] Weinberger NA, Kersting A, Riedel-Heller SG, Luck-Sikorski C (2017). Body dissatisfaction in individuals with obesity compared to normal-weight individuals: a systematic review and meta-analysis. Obesity Facts.

[74] Schwartz D, Brownell K, Cash T, Pruzinsky T (2002). Obesity and body image. Body image: a handbook of theory, research and clinical practice.

[75] Castellini G, Mannucci E, Mazzei C, Lo Sauro C, Faravelli C, Rotella CM, Ricca V (2010). Sexual function in obese women with and without binge eating disorder. J Sex Med.

[76] Kolotkin RL, Zunker C, Østbye T (2012). Sexual functioning and obesity: a review. Obesity.

[77] Larsen S, Wagner G, Heitmann BL (2007). Sexual function and obesity. Int J Obes.

[78] Smith A, Patrick K, Heywood W, Pitts M, Richters J, Shelley J, Simpson JM, Ryall R (2012). Body mass index, sexual difficulties and sexual satisfaction among people in regular heterosexual relationships: a population-based study. Int Med J.

[79] Yazdznpanahi Z, Beygi Z, Akbarzadeh M, Zare N (2016). Investigating the relationships between obesity and sexual function and its components. Shiraz E-Med J.

[80] Pujols Y, Meston CM, Seal BN (2010). The association between sexual satisfaction and body image in women. J Sex Med.

[81] Woertman L, van den Brink F (2012). Body image and female sexual functioning and behavior: a review. J Sex Res.

[82] Goodson P, Buhi ER, Dunsmore SC (2006). Self-Esteem and adolescent sexual behaviors, attitudes, and intentions: a systematic review. J Adolesc Health.

[83] Choi KB, Jang SH, Lee MY, Kim KH (2011). Sexual life and self-esteem in married elderly. Arch Gerontol Geriatr.

[84] Park SA, Kim MA (1999). The sexual life in climacteric women Korean. J Korean Acad Womens Health Nurs.

[85] Wiederman MW (1998). Volunteer bias in sexuality research using college student participants. J Sex Res.

[86] Borges R, Temido P, Sousa L, Azinhais P, Conceição P, Pereira B, Sobral F (2009). Metabolic syndrome and sexual (dys) function. J Sex Med.

[87] Corona G, Jannini E, Maggi M (2006). Inventories for male and female sexual dysfunctions. Int J Impot Res.

[88] Nappi RE (2007). New attitudes to sexuality in the menopause: clinical evaluation and diagnosis. Climacteric.

[89] Weaver AD, Byers ES (2006). The relationships among body image, body mass index, exercise, and sexual functioning in heterosexual women. Psychol Women Q.

